# Longitudinal relationships between diffusion and macrostructural Alzheimer's disease signatures

**DOI:** 10.1002/alz70856_101636

**Published:** 2025-12-25

**Authors:** McKenna E. Williams, Jeremy A. Elman, Tyler R. Bell, Christine Fennema‐Notestine, Carol E. Franz, Matthew S. Panizzon, Chandra A. Reynolds, Rongxiang Tang, William S. Kremen

**Affiliations:** ^1^ University of California San Diego, La Jolla, CA, USA; ^2^ University of Colorado Boulder, Boulder, CO, USA; ^3^ Texas A&M University, College Station, TX, USA

## Abstract

**Background:**

Diffusion‐based MRI metrics, such as gray matter mean diffusivity (gMD), may capture very early, microstructural Alzheimer‐related changes that predate macrostructural atrophy. We previously demonstrated that a novel Alzheimer's disease (AD) signature based on gMD can serve as a useful biomarker to predict future cognitive decline as early as middle age and can enhance identification of subgroups at risk for worse brain and cognitive outcomes when used alongside cortical thickness/volume signatures. This is crucial given evidence suggesting dynamic, biphasic changes in cortical thickness wherein higher, rather than lower, thickness is associated with very early AD pathology. For individuals on the AD spectrum, this pattern may be more likely among *APOE*‐ε4 carriers due to their elevated risk or it may just be observed earlier given forward shifting of their timeline of progression. Clarification of early trajectories in these AD signatures is needed to improve the specificity and sensitivity of these imaging biomarkers for identifying early AD‐related changes.

**Method:**

Participants were 286 men from 3 waves of the Vietnam Era Twin Study of Aging (VETSA; baseline age=56.36, SD=2.61; waves were ∼6 years apart). Multilevel models tested concurrent and lagged relationships between our previously published thickness/volume signature and the novel gMD signature that leverages the same regions and weightings. Models also tested whether longitudinal relationships between the AD signatures differed by *APOE*‐ε4 and cognitive status.

**Result:**

gMD and thickness/volume signatures showed negative concurrent relationships at all timepoints (*p*s < 0.001). Longitudinally, as expected higher (worse) gMD signatures predicted lower thickness/volume signatures 6 years later, even after accounting for concurrent associations between the signatures (β= ‐0.13, *p* = 0.023). In contrast, after accounting for concurrent relationships, *higher* thickness/volume signatures predicted higher (worse) gMD signatures (interaction β= 0.27, *p* = 0.014) and higher risk of MCI (OR=1.49, interaction *p* = 0.011) 6 years later, *but only among APOE‐ε4‐positive individuals*.

**Conclusion:**

The results support gMD signatures as early markers of AD‐related change that precede changes in thickness/volume. Findings are consistent with a biphasic model in which very early increased cortical thickness/volume (perhaps co‐occurring with increased gMD) in AD‐related regions, particularly among individuals with an *APOE*‐ε4 allele, may indicate risk for future decline.

